# The Relationship between Physical Activity, Self-Regulation and Cognitive School Readiness in Preschool Children

**DOI:** 10.3390/ijerph182211797

**Published:** 2021-11-10

**Authors:** Pulan Bai, Sarah Johnson, Stewart G. Trost, Leanne Lester, Andrea Nathan, Hayley Christian

**Affiliations:** 1 School of Population and Global Health, The University of Western Australia, Perth, WA 6009, Australia; hayley.christian@uwa.edu.au; 2 Telethon Kids Institute, The University of Western Australia, Perth, WA 6009, Australia; sarah.johnson@telethonkids.org.au (S.J.); andrea.nathan@telethonkids.org.au (A.N.); 3 School of Human Movement and Nutrition Sciences, The University of Queensland, Brisbane, QLD 4072, Australia; s.trost@uq.edu.au; 4 School of Human Sciences, The University of Western Australia, Perth, WA 6009, Australia; leanne.lester@uwa.edu.au

**Keywords:** preschooler, young children, child development, self-regulation, cognitive development

## Abstract

(1) Background: Limited research exists on the pathways through which physical activity influences cognitive development in the early years. This study examined the direct and indirect relationships between physical activity, self-regulation, and cognitive school readiness in preschool children. (2) Method: Participants (*n* = 56) aged 3–5 years were recruited from the PLAYCE study, Perth, Western Australia. Physical activity was measured using 7-day accelerometry. Self-regulation was measured using the Head Toes Knees and Shoulders task and cognitive school readiness was assessed using the Bracken School Readiness Assessment. Baron and Kenny’s method was used for mediation analysis. (3) Results: After adjustment for socio-demographic factors, total physical activity was positively and significantly associated with cognitive school readiness (B = 0.16, SE = 0.07, *p* ≤ 0.05). Moderate-vigorous physical activity (MVPA) was positively and significantly associated with self-regulation (B = 0.3, SE = 0.13, *p* ≤ 0.05) and cognitive school readiness score (B = 0.20, SE = 0.09, *p* ≤ 0.05). Self-regulation was found to be a partial mediator of the relationship between MVPA and cognitive school readiness. (4) Conclusion: These findings highlight the direct and indirect association between preschool children’s physical activity, self-regulation, and cognitive school readiness. Further research is needed to determine the causal relationships between young children’s physical activity and cognitive development, over time.

## 1. Introduction

Early childhood represents a critical period in brain growth and cognitive development [1]. The structure of the brain in young children is established through a series of dynamic interactions between environmental conditions and experiences [2]. Physical activity and active play in children provides an important pathway through which these interactions can occur [2]. However, many young children do not meet the recommended physical activity guidelines [3,4], which could potentially have a negative impact on a child’s physical, cognitive, social, spiritual and emotional growth [1]. The Canadian and Australian 24-h Movement Guidelines for the Early Years recommend children aged 1–5 years accumulate at least 180 min per day of physical activity. For children aged 3–5 years the Guidelines also recommend that at least 60 min of the recommended 180 min of physical activity per day is moderate-vigorous physical activity (MVPA) [5,6].

These guidelines highlight the importance of physical activity in preschool aged children, however, to date, studies exploring the link between physical activity and cognitive outcomes lacks consensus [7]. One possible reason for the mixed findings may be that studies using objective measures of physical activity do not consider if the relationship between physical activity and cognitive outcomes varies by the intensity of physical activity (e.g., MVPA vs. total physical activity). A deeper understanding of the type and intensity of physical activity that positively impacts developmental outcomes in preschool children will contribute towards a better understanding of the pathways through which physical activity influences a young child’s development, particularly their cognitive development [7]. Such information is needed to better inform the design of future physical activity intervention studies to better support young children’s cognitive development. This is important because developmental delays in cognitive and physical development may cause significant health, social and economic consequences in the later years of child and adult life which, in turn, exerts substantial financial and social burdens on society [1].

Cognitive development in the preschool years is crucial for future learning and academic achievement [8]. School readiness, as defined by the U.S. National Education Goals Panel (1995), consists of five major domains being: “Language development”, “Approaches towards learning”, “Physical well-being and motor development”, “Social and emotional development”, and “Cognition and general knowledge” [9]. Of the five domains, the cognitive and general knowledge domain, or what we refer to as the cognitive school readiness domain, is most important for a child’s school readiness since cognitive development supports self-regulation, onward problem solving, and information processing which are required for learning in school [10,11,12].

One potential mechanism or pathway through which physical activity may influence cognitive development is self-regulation. Physical activity is associated with the activation of the prefrontal cortex, the same part of the brain responsible for self-regulatory behaviours. Therefore, it is possible that physical activity directly influences self-regulation [13]. Self-regulation is a multi-dimensional construct. In this paper we define self-regulation as behavioural self-regulation, which includes attention control, working memory, and inhibitory control [14]. In children this includes the ability to control impulses, keep information in mind, maintain attention and apply information they have learned [15]. 

Research involving young children has shown a positive relationship between physical activity and behavioural self-regulation, however the evidence is limited. To our knowledge, only a few papers have investigated the relationship between physical activity and self-regulation in preschool children. Howard et al. reported that participation in individual sports at the age of 4 was associated with higher self-regulation than at age 6, compared to those who did not participate [16]. Similarly Becker et al. showed that active play may have a positive effect on preschool children’s self-regulation [17]. Kybartas et al. [18] also reported significant correlations between vigorous intensity physical activity levels and self-regulation in children aged from three to five years. Findings from these studies suggest a beneficial direct effect of physical activity on self-regulation in children, however they are limited because they do not adjust for potential confounders (e.g., child age and gender) and the findings are not generalizable to broader populations of young children. Further research is needed to address these methodological limitations.

In addition, further research is needed to explore the pathways through which physical activity influences preschool children’s cognitive development. Given that self-regulation skills underlie many of the attributes and behaviours associated with cognitive school readiness, and there is some evidence to show that higher self-regulation is associated with better school readiness outcomes [15,19], as well as some evidence that physical activity is associated with both self-regulation [17] and cognitive development [18], it is possible that self-regulation mediates the relationship between physical activity and cognitive school readiness. A recent systematic review on preschool children’s physical activity, school readiness, and cognition showed that there is still not a clear consensus of the evidence regarding the association between physical activity and preschool children’s cognitive outcomes [7]. The review reported mixed findings and called for more research to understand the mechanistic pathways between physical activity, self-regulation, and cognitive outcomes in preschool children. 

Thus, the aim of this study was to examine the direct and indirect relationship between self-regulation, cognitive school readiness and physical activity in preschool aged (3–5 years) children. We hypothesised that: (1) Higher levels of physical activity are associated with higher levels of self-regulation; (2) Higher levels of self-regulation are associated with higher levels of cognitive school readiness; (3) Higher levels of physical activity are associated with higher levels of cognitive school readiness; (4) Self-regulation is a possible mediator by showing a plausible reduction in a direct effect between physical activity and cognitive school readiness.

## 2. Materials and Methods

This cross-sectional study formed part of the Play Space & Environments for Children’s Physical Activity (PLAYCE) Study [20]. The PLAYCE Study (2015–2018) was a large cross-sectional observational study that measured the environmental influences on 1596 preschool children’s physical activity across different behavioural settings: long day-care centre, home, and the neighbourhood in metropolitan Perth, Western Australia [20,21]. Long day care centres were recruited, sampled, and segmented into quartiles based on the number of approved places at each centre to ensure that there was maximum variability in the physical environments between centres. Eligible centres were initially recruited and then parents of children aged 2–5 years from each recruited centre were invited to participate. Further details of the PLAYCE Study’s design and methods have been reported elsewhere [20]. 

### 2.1. Sample

Participants included children aged 3–5 years attending a long day-care centre in Perth, Western Australia. Study consent was provided by both centre directors and parents. Participants were recruited from those children who had already participated in the PLAYCE Study and who indicated in the parent survey that they would happy to participate in future PLAYCE related studies (*n* = 270). Of these, 123 were not eligible because they no longer met the age eligibility criteria (>5 years old) and/or no longer attended childcare. Another 23 parents could not be contacted. Thus, 123 parents and children were invited to participate of which 56 agreed (response rate 45%). Between 1 and 8 children were recruited from 18 long day care centres taking part in the PLAYCE study. Parents who agreed to participate in the current study provided further consent for their child to take part in the self-regulation and cognitive school readiness assessment. 

### 2.2. Measures

#### 2.2.1. Self-Regulation

Behavioural self-regulation was measured using the Head-Toes-Knees-Shoulders (HTKS) task [22] which is a widely used and reliable measure with a reported alpha of 0.98 for overall inter-rater reliability [22,23]. This measure has also been shown to be a valid measure for assessing children’s behavioural self-regulation [19,22,24]. The HTKS task measures children’s behavioural self-regulation in diverse populations. It is a 15 -minute activity whereby children are asked to play a game in which they must do the opposite of what the assessor says. A “practice” was administered before the formal HTKS to see if each child could identify and touch the correct part of their body before proceeding to the formal HTKS. The formal HTKS consists of two parts. In the first part, the assessor instructs children to touch their head (or their toes), but instead of following the command, the children are required to do the opposite and touch their toes. If children pass this part of the HTKS, they progress onto the second part, where the knees and shoulders commands are added. A score of zero denotes an incorrect response, one point is given for a self-corrected response and two points for a correct response. Higher HTKS scores indicate higher levels of behavioural regulation [22,23]. Raw scores from the HTKS task were converted to a percentage.

#### 2.2.2. Cognitive School Readiness

Cognitive school readiness was measured using the Bracken School Readiness Assessment—Third Edition (BSRA-3) [25]. BSRA-3 is a widely used measure for predicting young children’s school readiness [26] because of its adequate validity, ease of administration and instructional properties [25,26]. It is a highly reliable measure with test-retest stability coefficients ranging from 0.76 to 0.92 [25]. Colours, numbers, letters, sizes, shape recognition and comparison ability are assessed. The BSRA-3 takes approximately 15 min to complete and is administered by a trained assessor. The assessor shows the child a series of coloured stimuli pictures with numbers and asks the child to say or point to the number of the picture according to the assessor questions. Each question relates to a specific concept, and for each item in the test the child is asked to make a choice among 4 pictures except for the colour test which requires the child to identify 11 colours. Zero denotes an incorrect response and one is a correct response [27]. According to the BSRA-3 scoring guideline [27], the raw score was converted to a norm-referenced standard score. Higher BSRA-3 standard scores indicate better performance. The first author was trained in the use of the HTKS and BSRA-3 and administered all assessments.

#### 2.2.3. Physical Activity

Physical activity levels were measured using the ActiGraph GT3X+ accelerometer-based motion sensor (ActiGraph Corporation, Pensacola, FL, USA) [28]. This device has moderate-to-strong validity for measuring the amount and intensity of daily physical activity in preschool children [28]. Preschool children wore the accelerometer for seven consecutive days, as per standard protocol in physical activity research. Seven-day monitoring provides an accurate measure of physical activity in this age group and takes into account variation in physical activity between weekdays and weekends [29]. Established cut-points developed by Pate and colleagues were used to determine daily time spent in MVPA (420 counts/15 s for moderate physical activity and 842 counts/15 s for vigorous physical activity) as well as light intensity physical activity (LPA) (200 counts/15 s) so total physical activity (LMVPA) could be determined [30,31,32]. Sampling intervals (epochs) of 15-s were used to accommodate the typical physical activity behaviour of preschool children. Data validation was based on at least 480 min of daily wear time and participants were included in the analyses if they had four or more valid monitoring days, with at least three weekdays and one weekend day, and a minimum eight hours wear time/day. Non-wear time was defined as intervals with at least 20 consecutive minutes of 0 counts, with allowance for up to 2 min under the count threshold for sedentary activity. A diary was used by parents and carers to record the duration and reasons for the accelerometer being removed during the seven days and wear time was estimated by subtracting non-wear time from the total monitoring time for the day.

#### 2.2.4. Socio-Demographic Factors

Child age, gender, parent education (No formal qualification; Year 10 or equivalent; Year 12 or equivalent; Trade/apprenticeship/certificate; Diploma; University degree; Post-graduate qualification) and residential address were collected as part of the PLAYCE parent survey [20]. Residential socio-economic status (SES) of residential suburb was measured using the Australian Bureau of Statistics Socio-Economic Indexes for Areas (SEIFA) suburb ranking score from the Australian Index of Relative Socio-Economic Advantage and Disadvantage (IRSAD) [33]. Scores were ranked and split into three tertiles of low, medium, and high SES.

### 2.3. Statistical Analyses

Initially, confounders were identified through bivariate correlation analysis. Child age has been shown in previous studies [17] to be positively correlated with active play and self-regulation, thus was automatically included as a confounder. SES and child gender were also included in the linear regression models as bivariate correlation and multilevel regression analyses showed that they were significantly associated with the independent variable (physical activity) and dependent variables (self-regulation and cognitive school readiness score). Parental education level was not included as it was not significantly related to the independent or dependent variables.

Multilevel mixed-effect regression analyses with long day care centre as a random effect were conducted to examine the effects of physical activity (total physical activity and MVPA) on self-regulation and cognitive school readiness, while controlling for children’s age, gender, and SES. The multi-level mixed model accounted for the possibility that responses from individuals (first level) at the same day care centre may be more similar than responses from a different centre (second level), that is, a clustered random effect. All of the first level variables were fixed effects.

Using methods originally outlined by Baron and Kenny [34], four regression models were run to test for the possible mediation effect of self–regulation on the relationship between physical activity (total physical activity and MVPA) and cognitive school readiness: (1) simple regression analysis with physical activity predicting self-regulation; (2) simple regression analysis with physical activity predicting cognitive school readiness; (3) simple regression analysis with self-regulation predicting cognitive school readiness; (4) multiple regression analysis with physical activity and self-regulation predicting cognitive school readiness. If all of the associations from the first three regression models were significant and if in the fourth regression model physical activity was no longer significant when self-regulation was controlled for, this would support full mediation. However, if physical activity was still significant, this would support partial mediation. The mediation relationship was also tested using a bootstrap estimation approach sampling the dataset with replacement using 50 samples to detect if the sampling distribution of the mediated effect was skewed away from 0. If zero did not occur between the lower limit and upper limit of the confidence interval, this would demonstrate significant indirect mediation effect [35,36].

## 3. Results

### 3.1. Sample Characteristics

The mean age of children was 3.5 years (±Standard Deviation (SD) 0.69) and 54% were male. Almost all (96%) of the parents were female, 66% had a bachelor’s degree or higher, and 55% lived in a high socioeconomic suburb (Table 1). On average, children accumulated 162 min/day (±SD 37.7) of total physical activity and 80 min/day of MVPA (±SD 28.6). The mean self-regulation score was 56% (±SD 28.5) and the age adjusted cognitive school readiness mean standard score was 109 (±SD 15.3).

### 3.2. Relationship between Physical Activity, Self-Regulation, and Cognitive School Readiness

After adjustment for socio-demographic factors and child-centre clustering effects, daily MVPA was significantly and positively associated with self-regulation and cognitive school readiness (Table 2). For every minute increase in MVPA, there was a 0.29% increase in the self-regulation score (SE = 0.13, *p* = 0.03) and 0.20 unit increase in the cognitive school readiness score (SE = 0.09, *p* = 0.02). Self-regulation and cognitive school readiness scores were also positively associated with each other (SE = 0.08, *p* = 0.009); for every percentage increase in the self-regulation score, there was a 0.22 increase in the cognitive school readiness score.

Associations between total physical activity and self-regulation and cognitive school readiness score were similar to those found for MVPA. Total physical activity was positively and significantly associated with cognitive school readiness (B = 0.16, SE = 0.07, *p* ≤ 0.05). Overall, however, the effect sizes were smaller and the association between total physical activity and self-regulation was not quite statistically significant (B = 0.18, SE = 0.10, *p* = 0.06) (Table 2).

### 3.3. Mediation Relationship between Physical Activity, Self-Regulation and Cognitive School Readiness

After adjusting for sociodemographic factors and clustering effects, total physical activity was significantly associated with self-regulation (B = 0.22, SE = 0.08, *p* = 0.00), however, self-regulation was not significantly associated with cognitive school readiness (B = 0.19, SE = 0.10, *p* = 0.07), suggesting that it is unlikely that self-regulation mediates the relationship between total physical activity and cognitive school readiness.

**Figure 1:**
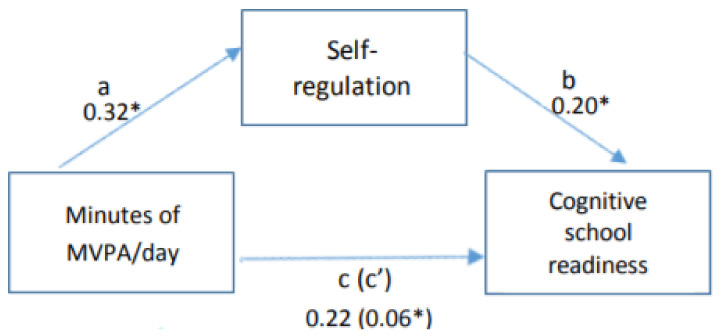
Relationship between minutes of MVPA per day, self-regulation and cognitive school readiness (adjusted for age, gender, home suburb SES and centre clustering effects). * *p* < 0.05.

After adjusting for sociodemographic factors and clustering effects, MVPA was significantly associated with self-regulation (pathway a; B = 0.32, SE = 0.11, *p* < 0.01), self-regulation was significantly associated with cognitive school readiness (pathway b; B = 0.20, SE = 0.08, *p* = 0.01), however MVPA was no longer significantly associated with cognitive school readiness after controlling for the possible mediator self-regulation (pathway c; B = 0.22, SE = 0.13, *p* = 0.06) (Figure 1). After applying the bootstrap estimation approach the direct co-efficient was still significant (pathway c; B = 0.06, SE = 0.03, *p* = 0.03). According to Baron and Kenny’s mediation hypothesis [34], this indicates that self-regulation is a possible partial mediator of the association between MVPA and cognitive school readiness.

## 4. Discussion

Significant positive associations between preschool children’s MVPA and self-regulation were observed in the adjusted models. This finding is consistent with other studies examining the relationship between objectively measured preschool children’s physical activity and self-regulation using the HTKS [17,18]. However, previous studies were limited as they did not control for known correlates of preschool children’s physical activity (i.e., child gender, age and SES) or focused on a specific type of physical activity only (i.e., individual sport participation) [16]. Our study provides stronger evidence of a positive association between preschool children’s MVPA and self-regulation.

This study also found a significant positive association between preschool children’s MVPA and cognitive school readiness. For every additional minute of MVPA preschool children did per day, their cognitive school readiness score increased by 0.2 and their self-regulation score increase by 0.3%. According to McClelland et al., this increase in the self-regulation score is equivalent to the learning gain in vocabulary made in about two days of pre-kindergarten [19]. Depending on a child’s age and score quartile, a percentage increase in the self-regulation score could increase a child’s cognitive school readiness score by 0.2 [25]. This represents a small but statistically significant increase in cognitive school readiness. For children aged 3–5 years the Canadian and Australian 24-Hour Movement Guidelines recommend children accumulate at least 60 min/day of energetic play (or MVPA) per day [5,6]. Given that, in many countries, a large proportion of children fail to meet this guideline increasing young children’s MVPA could have significant health as well as cognitive developmental benefits. 

In contrast to our findings, other studies have found no significant association between preschool children’s MVPA and cognitive school readiness when only specific components (i.e., emergent literacy and math achievement) of cognitive school readiness were measured [17,37]. It is possible that our more comprehensive overall measure of cognitive school readiness (the BSRA-3) [25] enabled the relationship between preschool children’s physical activity and other important components of cognitive school readiness (e.g., ability to identify and compare shapes and sizes) to be identified. Further research is needed to provide a deeper understanding of the relationship between preschool children’s MVPA and different components of cognitive school readiness and use longitudinal designs to confirm the direction of the causal relationships between physical activity and cognitive school readiness.

A unique finding of this study relates to the relationship between the intensity of physical activity and young children’s self-regulation and cognitive school readiness. Both MVPA and total physical activity were positively associated with cognitive school readiness, however only MVPA was positively associated with self-regulation and, overall, the effect sizes were greater for MVPA and self-regulation and cognitive school readiness. Our findings suggest that higher intensity physical activity may be more important for promoting self-regulation and cognitive school readiness in preschool children. Past research has suggested that MVPA may promote the development of anterior frontal brain patterns which is important for cognitive control [38], and this may explain why MVPA rather than total physical activity was significantly associated with self-regulation and cognitive school readiness.

Finally, our findings showed that self-regulation was a partial mediator of the relationship between MVPA, but not total physical activity, and cognitive school readiness. When self-regulation was added to the model of the association between MVPA and cognitive school readiness, the association attenuated but still was significant. This finding is consistent with Becker et al.’s study which identified self-regulation as a mediator between MPVA and cognitive outcomes [17]. These findings combined suggest that MVPA may have a positive indirect effect on preschool children’s cognitive school readiness by enhancing self-regulation. Physical activity intervention studies aimed at improving young children’s self-regulation and measuring the effect on outcomes such as cognitive school readiness are warranted. 

This study was limited by its cross-sectional design and thus causal relationships between physical activity, self-regulation and cognitive school readiness could not be examined. Future research should use a longitudinal observational study design to establish the temporal sequence and causal relationships, as well as the impact on children’s future success. In addition, further research is warranted to confirm if the findings from this study are apparent in older-school aged children.

The small sample size and higher SES of parents limits the generalisability of the findings from this study. However, it is important to note that SES has not been identified as a correlate of preschool children’s physical activity [39,40]. Future research should consider if socio-demographic variables moderate the mediation relationships found. 

A strength of this study was the use of validated measures of self-regulation, cognitive school readiness and seven-day monitoring of objectively measured physical activity. Furthermore, data were collected from 18 childcare centres which reduced the impact of clustering effects and provided more variation in the data collected.

## 5. Conclusions

In conclusion, our findings show that physical activity is positively associated with self-regulation and cognitive school readiness in preschool children. This highlights that it is important to promote physical activity within this young age group, not only for health reasons but also to support their cognitive development and school readiness. Evidence-based strategies to increase physical activity levels in preschool children are required as well as the evaluation of the impact of such interventions on health and developmental outcomes in young children. These findings contribute to the evidence base that physical activity not only provides health benefits for young children but is important for the development of the whole child, which includes their physical, social-emotional, and cognitive development. 

## Figures and Tables

**Table 1:** Characteristics of study sample.

	*n*	% Or Mean (SD)
Socio-demographics		
Child gender (male)	56	53.60
Child mean age	56	3.45 (0.69)
Parent gender (female) ^1^	56	96.43
Parent highest education level ^1^		
Secondary or less	11	19.60
Trade/apprenticeship/certificate	8	14.30
Bachelor degree or higher	37	66.10
SES ^2^	56	
Low	4	7.10
Medium	21	37.50
High	31	55.40
Physical Activity Levels		
Total physical activity(min/day) ^3^	56	161.67 (37.73)
MVPA (min/day) ^3^	52	79.99 (28.63)
Cognitive Outcomes		
Self-regulation ^4^	55	55.75 (28.46)
Cognitive school readiness ^5^	56	109.07 (15.30)

^1^ For parents who completed the survey; ^2^ SES of residential suburb by SEIFA State Suburb Code (SSC) Index of Relative Socio-Economic Advantage and Disadvantage (IRSAD). Decile ranking score split into tertiles; ^3^ Average minutes per day of physical activity were measured by seven-day accelerometry (Actigraph GT3X+); ^4^ Self-regulation measured using the Head, Toes, Knees and Shoulders test (score measured as a%). Higher score indicates better performance; ^5^ Age-standardised cognitive school readiness standard score calculated using the BSRA-3 scoring guideline (possible score range of 40–160). Higher score indicates better performance.

**Table 2:** Association between MVPA, total physical activity, self-regulation score and cognitive school readiness.

	Model 1 Self Regulation ^1^	*p* Value	Model 2-Cognitive School Readiness ^2^	*p* Value	Model 3-Cognitive School Readiness ^3^	*p* Value
	B (SE)		B (SE)		B (SE)	
Child age (years)	17.46 (4.91)	<0.01 **	−1.73 (3.41)	0.61	−3.22 (3.66)	0.378
Child gender (male ^)	3.04 (7.21)	0.67	5.10 (4.71)	0.28	1.37 (3.79)	0.717
Mid SES ^4^	22.62 (11.99)	0.06	0.15 (8.97)	0.99	−4.29 (9.09)	0.637
High SES ^4^	32.15 (12.01)	<0.01 **	9.65 (9.15)	0.29	−0.16 (9.21)	0.989
Mins of MVPA/day	0.29 (0.13)	0.03 *	0.20 (0.09)	0.02 *	-	-
Mins of total physical activity/day	0.18 (0.10)	0.06	0.16 (0.07)	0.02 *	-	-
Self-regulation	-	-	-	-	0.22(0.08)	0.009 **

All models controlled for child age, gender, and centre SES’; * *p* < 0.05, ** *p* < 0.01—variable not included in the analysis; ^ Reference category is male; ^1^ Relationship between self-regulation and physical activity; ^2^ Relationship between cognitive school readiness and physical activity; ^3^ Relationship between self-regulation and cognitive school readiness; ^4^ Reference group = low SES. SES by SEIFA State Suburb Code (SSC) Index of Relative Socio-Economic Advantage and Disadvantage (IRSAD). Decile ranking score split into tertiles.

## Data Availability

The data presented in this study are available on request from the corresponding author.
